# Evaluating the impact of COVID-19 on the HIV epidemic among MSM in Australia

**DOI:** 10.1097/QAD.0000000000004344

**Published:** 2025-09-15

**Authors:** Rongxing Weng, Jisoo A. Kwon, Mo Hammoud, Brent Clifton, Nick Scott, Skye McGregor, Richard T. Gray

**Affiliations:** a Kirby Institute, UNSW Sydney; b National Association of People With HIV Australia, Sydney, New South Wales; c Burnet Institute, Melbourne, Victoria, Australia.

**Keywords:** Australia, COVID-19, HIV, modeling, MSM

## Abstract

**Objective:**

Government-imposed physical distancing restrictions during the COVID-19 pandemic disrupted biobehavioral HIV prevention practices and access to healthcare services. This study aimed to use a mathematical model to evaluate the impact of COVID-19 on the HIV epidemic among MSM in Australia, using empirical data.

**Design:**

A retrospective modeling study.

**Methods:**

We developed a mathematical model to estimate monthly HIV incidence between January 2020 and August 2022. We obtained aggregated monthly data for sexual partners, condom use, HIV testing, preexposure prophylaxis (PrEP) use, and migration. Three scenarios were simulated: a COVID-19 scenario; a no COVID-19 scenario where input parameters remained at pre-COVID-19 values; and a no COVID-19 scenario with continued PrEP scale-up.

**Results:**

In the absence of the COVID-19 pandemic, 1263 (95% percentile interval: 880–1706) infections would have occurred between January 2020 and August 2022 compared to 915 (95% percentile interval: 638–1282) for the COVID-19 scenario (a 27.6% reduction). Reduced sexual partners was the leading factor contributing to the change in HIV infections and diagnoses (-24.9 and -10.6%, respectively). MSM aged at least 50 years had a larger reduction (31.0%) in new HIV infections than their younger counterparts (19.9%).

**Conclusion:**

A substantial reduction in new HIV infections and diagnoses in Australia occurred during the COVID-19 pandemic, largely due to decreased numbers of sexual partners. This reduction underscores the need for sustained public health strategies leveraging reduced transmission rates to continue progress toward eliminating HIV in Australia.

## Introduction

The coronavirus disease 2019 (COVID-19) pandemic caused widespread disruption to healthcare systems worldwide [1]. This resulted in reduced clinic visits, testing, and treatment for infectious diseases, including the HIV, potentially increasing their burden [2]. In Australia, early public health responses included lockdowns, border closures, and physical distancing measures [3,4], followed by a vaccine rollout from February 2021 [5]. Most restrictions were lifted in December 2021 when over 90% of the adult population were fully vaccinated [6], and international borders were reopened in February 2022 [7].

These measures significantly affected sexual behaviors and HIV-related services in Australia, with reported declines in HIV testing and sexual contacts during lockdowns [3,4,8]. Although home-based HIV testing was available during the pandemic period, its uptake was limited [9]. Prevention behaviors were also affected. In 2020, 42% of MSM discontinued preexposure prophylaxis (PrEP) [10], and similar trends were observed internationally [11]. These factors likely contributed to a 50% reduction in HIV diagnoses, from 895 in 2019 to 541 in 2021 [12], but their contribution to this reduction is unknown and the long-term impact of these changes on the HIV epidemic remains uncertain, highlighting the need for a comprehensive evaluation.

While modeling studies have assessed the effects of COVID-19 on HIV transmission [13–16], many relied on assumed changes to HIV transmission factors, rather than directly observed impacts. Most modeling studies assumed constant impacts and often overlooked the dynamic effects of specific policies such as lockdowns and border closures [13–16]. More nuanced studies that examine the dynamics of the HIV epidemic in response to COVID-19 control measures are needed.

MSM are one of the key populations affected by HIV [17]. In Australia, MSM account for 70% of new HIV diagnoses [12], with around 90% of infections linked to nonromantic partners (casual sexual partners and friends with benefits) [18]. Detailed datasets are available for this population in Australia and include monthly changes in sexual behaviors, HIV testing, and migration during the acute phase of the COVID-19 pandemic. This study aimed to use this empirical data within a mathematical model to evaluate the impact of COVID-19 restrictions on the HIV epidemic among MSM in Australia.

## Materials and methods

We developed a simple but general and flexible HIV transmission model which we then applied to the MSM population in Australia. This Simple HIV Model is written in R version 4.3.2. and is publicly available [19]. This study was approved by the UNSW Sydney Human Research Ethics Committee (iRECS6381). De-identified and aggregated data on the number of monthly HIV diagnoses were sourced from the Australian National HIV Registry surveillance data. Consent was not sought from those diagnosed with HIV as the data were collected under routine HIV surveillance protocols.

### Simple HIV model

Our model expands previously developed models [20–22]. It uses difference equations to describe the HIV cascade and the effect of changes in sexual partners, condom use, HIV testing, and PrEP use on HIV transmission. At a prespecified timestep *t*, *N*_*t*_ is the number of people living with HIV in the population, *I*_*t*_ is the number of new infections, and *β*_*t*_ is the transmission probability from a person with an unsuppressed HIV viral load to a susceptible partner not on PrEP. If there were no interventions, HIV-positive individuals would transmit *N*_*t*_*β*_*t*_ infections at timestep *t*. To represent the HIV cascade, we defined *d*_*t*_ to be the proportion of people with HIV who are diagnosed, *τ*_*t*_ to be the proportion of those diagnosed on antiretroviral therapy (ART), *σ*_*t*_ to be the proportion of those on ART with suppressed viral load (< 200 copies per ml). If *ϕ* is the reduction in transmission due to viral suppression, then HIV-positive individuals transmit *N*_*t*_*β*_*t*_ (1-*d*_*t*_*τ*_*t*_*σ*_*t*_*ϕ*) infections at timestep *t*. Defining *ω*_*t*_ to be the proportion of the susceptible population on PrEP, and *ε*_*p*_ to be the efficacy of PrEP in preventing infection, the number of new infections at timestep t due to transmission to susceptible sexual partners is then given by:


(1) $$I_{t}=N_{t}\beta_{t}\left(1-d_{t}\tau_{t}\sigma_{t}\phi \right)\left(1-\omega_{t}\varepsilon_{p}\right)$$

To account for migration and mortality, we defined *M*_*t*_ to be the number of people who enter Australia living with HIV, *E*_*t*_ to be the number of people who leave Australia living with HIV, and *μ*_*t*_ to be the probability of all-cause mortality among people with HIV. Then the number of people living with HIV in the population at timestep *t*+1 would be:


(2) $$N_{t+1}=\left(N_{t}+I_{t}+M_{t}-E_{t}\right)\left(1-\mu_{t}\right)$$

Before calculating *N*_*t*_ and *I*_*t*_, a value for the transmission probability (*β*_0_) at time *t* = 0 is required. Using initial values for the population size, new infections, and each parameter in Equation 1:


(3) $$\beta_{0}=I_{0}/\left(N_{0}\left(1-d_{0}\tau_{0}\sigma_{0}\phi \right)\left(1-\omega_{0}\varepsilon_{p}\right)\right)$$

### Effect of changing sexual behaviors

The impact of changes in sexual behaviors is captured through condom use and sexual partners. If the proportion of condom use changes to *c*_*t*_ at timestep *t* compared to an initial level *c*_0_ with an effectiveness of *ε*_*c*_, the relative change in the transmission probability is given by $\frac{1-\varepsilon_{c}c_{t}}{1-\varepsilon_{c}c_{0}}$. If the number of sexual partners changes to *p*_*t*_ compared to a pre-COVID level of *p*_0_, the relative change in the transmission probability is given by $\frac{p_{t}}{p_{0}}$. By applying these relative changes to Equation 3, we obtain:


(4) $$\beta_{t}=\left(\frac{p_{t}}{p_{0}}\right)\left(\frac{1-\varepsilon_{c}c_{t}}{1-\varepsilon_{c}c_{0}}\right)\beta_{0}$$

### Effect of changes in testing rates

The proportion diagnosed over time is also affected by changes in testing rates. If the proportion *T*_*t*_ of the population is tested, the number of people living with HIV diagnosed at the next timestep is given by:


(5) $$N_{t+1}d_{t+1}=N_{t}d_{t}+I_{t}T_{t}+\left(1-d_{t}\right)N_{t}T_{t}$$

With Equation 2, this can be used to calculate the proportion diagnosed:


(6) $$d_{t+1}=\frac{N_{t}d_{t}+\left[I_{t}+\left(1-d_{t}\right)N_{t}\right]T_{t}}{\left(N_{t}+I_{t}+M_{t}-E_{t}\right)\left(1-\mu_{t}\right)}$$

We only included diagnosed immigrants ($M_{t}$) and emigrants ($E_{t}$) in the model assuming the number undiagnosed is relatively small and will have a minimal impact on the results.

Defining the number of new diagnoses at the initial timestep to be *D*_0_, the proportion of the population tested at the initial timestep (*T*_0_) can be calculated and then used to calculate *d*_*t*+1_:


(7) $$T_{0}=D_{0}/\left(I_{0}+\left(1-d_{0}\right)N_{0}\right)$$

### Application of simple HIV model

We focused on the MSM population in Australia using a monthly timestep starting from December 2019. Notifications were used for the initial number of diagnosed cases (*D*_0_) [12]. Changes in each parameter were captured following the implementation of restrictions and border closures in response to COVID-19 in March--April 2020 [23]. For January--February 2020, we assumed parameters remained at their December 2019 values. We used the reported number of nonromantic partners as a proxy for the number of sexual partners at each time step, and the reported proportion of MSM with casual partners reporting no condomless anal intercourse in the past 4 weeks as a proxy for condom use. Constant parameters are presented in Table 1, with the time-varying variables *ω*_*t*_, *T*_*t*_, *c*_*t*_, and *p*_*t*_ (Table S1) estimated using data from monitoring of PrEP uptake in Australia, the Flux cohort study, and the Gay Community Periodic Survey [4,9,24,25] (details provided in the Supplementary Material).

**Table 1:** Constant input parameters and their associated descriptions and values (with uncertainty bounds) used in the model.

Parameter	Description	Value	Year	Uncertainty range	References and notes
Demographic					
*N*_0_	Number of MSM in Australia with HIV at the end of 2019	20 700		(18 534–25 748)	[12]^a^
*I*_0_	Number of new HIV infections among Australian MSM in the last month (December) of 2019	42		(38–49)	[12,30]^a^^,^^b^
*D*_0_	Number of new diagnoses among Australian MSM in the last month (December) of 2019	64		(50–77)	[12]^c^
*M*_0_	Number of MSM who enter Australia living with HIV in the last month (December) of 2019	15		(4–19)	[12]^d^
*E*_*t*_	Number of MSM who leave Australia living with HIV per month each year	9	2019	(0–17)	[12]^a^
		4	2020	(0–9)	
		6	2021	(0–11)	
		9	2022	(0–18)	
*μ*	Monthly all-cause mortality rate among MSM living with HIV (per 100 person-months)	0.068	2019	(0.039–0.117)	[12]^a^
		0.058	2020	(0.033–0.100)	
		0.096	2021	(0.055–0.165)	
		0.083	2022	(0.047–0.142)	
*Age*	Proportion of MSM aged <50 years in the Flux Study	0.598			[24]
	Proportion of MSM aged ≥50 years in the Flux Study	0.402			
Clinical					
*d*_0_	Proportion of all MSM with diagnosed HIV at the end of 2019	0.917		±5%	[12]^a^
*τ*	Proportion of MSM with diagnosed HIV taking ART at the end of each year	0.933	2019	±2.5%	[12]^a^
		0.938	2020	±2.5%	
		0.942	2021	±2.5%	
		0.951	2022	±2.5%	
*σ*	Proportion of MSM taking ART with suppressed virus at the end of each year	0.970	2019	±1.25%	[12]^a^
		0.965	2020	±1.25%	
		0.979	2021	±1.25%	
		0.984	2022	±1.25%	
*ω*_0_	Proportion of HIV-negative MSM who are on PrEP at the end of 2019	0.365		(0.345–0.386)	[9]^e^
HIV transmission					
*β*_0_	Probability of an MSM with untreated infection transmitting to a partner not on PrEP at the end of 2019	Calibration parameter			^f^
*ϕ*	Efficacy of ART in preventing HIV transmission if virus is suppressed	0.96		(0.74–0.99)	[29]^g^
*ε*_*p*_	Efficacy of PrEP in preventing HIV transmission (assuming 100% adherence)	0.86		(0.64–0.96)	[28]^g^
*ε*_*c*_	Efficacy of condoms when consistently used	0.705		(0.582–0.792)	[27]^g^
HIV related behaviors					
*T*_0_	Proportion of MSM tested per month at the end of 2019	Calibration parameter		±5%	^h^
*C*_0_	Proportion of MSM with casual partners reporting no condomless anal intercourse in the past four weeks at the end of 2019	0.731		(0.599–0.893)	[9,24]^i^
*p*_*t*_/*p*_0_	Average of monthly relative change in the number of sexual partners among MSM in the Flux Study from May 2020 to August 2022				[24]
	Overall	0.792			
	Aged <50	0.851			
	Aged ≥50	0.731			
*T*_*t*_/*T*_0_	Average of monthly relative change in the proportion of MSM tested in the Flux Study from May 2020 to August 2022				[24]
	Overall	0.862			
	Aged <50	0.946			
	Aged ≥50	0.731			

The uncertainty ranges of the parameters *d*_0_, *τ*, *σ*, and *T*_0_ were assumed (expressed as relative changes around each data point), as no empirical estimates of uncertainty were available. In general, a relative ± 5% range was applied, but when the upper bound of a proportion parameter would exceed 1, narrower ranges of ± 2.5% or ± 1.25% were applied instead.

ART, antiretroviral therapy; PrEP, preexposure prophylaxis.

a Estimates and their uncertainty ranges were generated using the methodology for producing the Australian HIV cascade for national surveillance [12], except for the uncertainty ranges of the parameters *d*_0_, *τ*, and *σ*.

b The number of new HIV infections among Australian MSM in the last month (December) of 2019, along with its uncertainty range, was determined by calculating the average monthly number of annual new infections, which was estimated from the European Centre for Disease Control (ECDC) model [30].

c Data on the number of monthly HIV diagnoses were sourced from the Australian National HIV Registry surveillance data. The number of new HIV diagnoses among Australian MSM in the last month (December) of 2019 was determined by calculating the average monthly number of annual new diagnoses. The associated uncertainty range was derived from the observed range of monthly number of diagnoses in 2019.

d The number of MSM who enter Australia already living with HIV in the last month (December) of 2019 was estimated by calculating the average monthly number of notifications attributed to male-to-male sex where the notified person had previously been diagnosed overseas in 2019. The associated uncertainty range was derived from the observed range of monthly number of notifications attributed to male-to-male sex where the notified person had previously been diagnosed overseas in 2019.

e The uncertainty range of proportion of HIV-negative MSM who are on PrEP at the end of 2019 was determined by calculating the corresponding 95% confidence interval using data from the Gay Community Periodic Survey [9].

f The probability of an MSM living with HIV who is undiagnosed and untreated transmitting HIV to a partner not on PrEP at the end of 2019 was calibrated to fit the average monthly number of new HIV infections among MSM in 2019 (*I*_0_).

g The uncertainty range was derived from the corresponding 95% confidence interval [27–29].

h The proportion of MSM tested per month at the end of 2019 was calibrated to fit both the average monthly number of new HIV infections among MSM in 2019 and the average monthly number of MSM diagnosed with HIV in 2019.

i We assumed a weekly declining trend (determined through a Poisson regression) in the proportion of MSM with casual partners reporting no condomless anal intercourse. The proportion of MSM with casual partners reporting no condomless anal intercourse in the past four weeks was calculated based on the corresponding behavior in the past week and in the past 6 months, which were reported in the Gay Community Periodic Survey and the Following Lives Undergoing Change (Flux) cohort study. The associated uncertainty range was derived from the Poisson regression.

### Scenarios

We simulated three scenarios from December 2019 to August 2022: the “COVID-19 scenario” incorporated all changes in monthly parameter values during the COVID-19 period; a counterfactual “no COVID-19 scenario,” where input parameters remained at their December 2019 value; and the “no COVID-19 plus PrEP scenario,” an alternative counterfactual scenario where PrEP scale-up and the corresponding decrease in condom use prior to 2019 continued during 2020–2022 (with other parameters remaining at their December 2019 values). In this scenario, PrEP coverage increased from 36.5 to 58.2% [25] and condom use decreased from 73.1 to 64.9% as per Gay Community Periodic Survey [9]. Further scenario details are provided in the Supplementary Material.

Simulations were also conducted to assess the individual effects of each time-varying parameter on the HIV epidemic. Age-stratified analyses using data from the Flux study focusing on younger (<50 years) and older (≥50 years) MSM were also conducted. This age cut-off was selected because different patterns in sexual behaviors and testing occur around this threshold [26] and smaller age categories had insufficient sample sizes for some time points. Migration was considered as a single variable incorporating both MSM living with HIV who enter (*M*_*t*_) and leave (*E*_*t*_) Australia.

### Model calibration, validation, and uncertainty analysis

We calibrated the initial transmission probability (*β*_0_) and testing rate (*T*_0_) to fit the average monthly number of new HIV infections and diagnoses among MSM in 2019 using Eqs. (3) and (7) [12]. The monthly numbers of MSM diagnosed with HIV from 2020 to 2022 were then used to validate the results of the model.

To account for uncertainty, all three scenarios were simulated 1000 times using randomly sampled parameters. The uncertainty ranges were mostly derived by calculating or using reported 95% confidence intervals [9,12,24,27–30]. For *M*_0_ and *D*_0_, the uncertainty corresponded to the observed range in their values over 2019. Uncertainty ranges were assumed for *d*_0_, *τ*, *σ*, and *T*_0_, as no empirical estimates of uncertainty were available. All parameter values at the initial timestep were drawn using uniform distributions. For time-varying parameters, values were also uniformly sampled at the final timestep, with intermediate values calculated using linear interpolation. The results from each simulation were then used to derive the 95% percentile interval, defined as the 2.5th and 97.5th percentiles, for the model outputs.

## Results

**Fig. 1:**
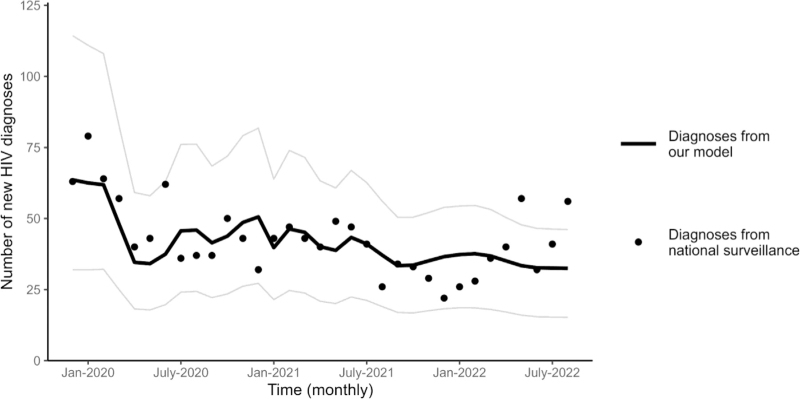
Comparison of estimated diagnoses from the model with diagnoses from national surveillance data.

After calibration to the December 2019 data, the estimated number of monthly diagnoses in the COVID-19 scenario closely aligned with data from national surveillance between January 2020 and August 2022 (Fig. 1), providing a validation for our model estimates.

Bands indicate 95% percentile intervals.

### Number of new infections, diagnoses, and MSM with HIV in each scenario

**Fig. 2:**
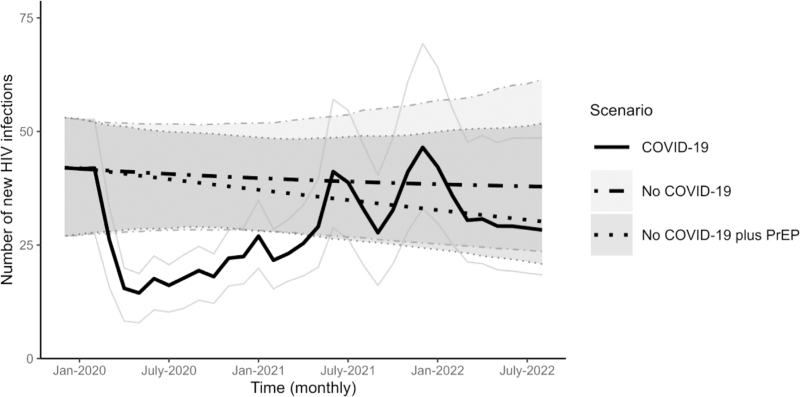
Monthly new HIV infections among men who have sex with men in Australia for each scenario.

In the no COVID-19 scenario, the estimated number of cumulative infections from 2020 to 2022 would have been the highest, at 1263 (95% percentile interval: 880–1706) (Table 2). Figure 2 shows that there was a stable level of monthly new infections with a slightly decreasing trend. In contrast, there was a 27.6% reduction (95% percentile interval: 22.8%–29.9%) in the number of cumulative infections for the COVID-19 scenario (915, 95% percentile interval: 638–1282) (Table 2). The largest reduction in infections (44.2%, 95% percentile interval: 43.1%–48.1%) occurred in 2020 with 273 (95% percentile interval: 179–347) infections versus 489 (95% percentile interval: 340–621) for the no COVID-19 scenario. There was a rebound with 387 infections (95% percentile interval: 266–546) in 2021, coinciding with a rebound in sexual partners (see Supplementary Material Figure S1), followed by a reduction to a stable level by August 2022 (Fig. 2). An increase in PrEP use (no COVID-19 plus PrEP scenario) would have resulted in a slight reduction in infections compared to the no COVID-19 scenario, but not as substantial as observed in the COVID-19 scenario. The estimated number of cumulative infections from 2020 to 2022 in the no COVID-19 plus PrEP scenario was 1149 (95% percentile interval PI: 872–1548), with a 9.0% reduction (95% percentile interval: -1.3 to 12.9) compared to the no COVID-19 scenario (Table 2). The monthly difference in the number of new infections between the no COVID-19 scenario and the no COVID-19 plus PrEP scenario expanded over time, with the largest difference in infections (21.1%, 95% percentile interval: 0.3–28.0) occurring in August 2022 (Fig. 2, Table S2). The uncertainty ranges for the three scenarios started to overlap in December 2020.

**Table 2:** Number of cumulative infections, cumulative diagnoses, and MSM with HIV in each scenario between January 2020 and August 2022.

Variables	Value	95% PI	Relative change	95% PI
Cumulative infections				
COVID-19	915	(638–1282)	−27.6%	(−29.9 to −22.8%)
No COVID-19	1263	(880–1706)	Reference	
No COVID-19 plus PrEP	1149	(872–1548)	−9.0%	(−12.9 to 1.3%)
Cumulative diagnoses				
COVID-19	1303	(689–2049)	−18.7%	(−26.5 to −13.8%)
No COVID-19	1603	(921–2414)	Reference	
No COVID-19 plus PrEP	1569	(903–2382)	−2.1%	(−4.3% to 0.4%)
MSM with HIV				
COVID-19	21 269	(19 345–26 003)	−2.0%	(−2.5 to −1.2%)
No COVID-19	21 696	(19 690–26 446)	Reference	
No COVID-19 plus PrEP	21 590	(19 617–26 329)	−0.5%	(−0.8 to 0.1%)

PI, percentile interval; PrEP, preexposure prophylaxis.

Bands indicate 95% percentile intervals.

Similarly, in the no COVID-19 scenario, the number of cumulative diagnoses over the simulation period would have been 1603 (95% percentile interval: 921–2414) compared to 1303 (95% percentile interval: 689–2049) for the COVID-19 scenario (an 18.7% reduction, 95% percentile interval: 13.8–26.5). The projected number of cumulative diagnoses in the no COVID-19 plus PrEP scenario was intermediate to the estimates of the other two scenarios, at 1569 (95% percentile interval: 903–2382) (Table 2). The number of MSM with HIV would have been slightly higher in the no COVID-19 scenario than that in the COVID-19 scenario, increasing from 21 269 (95% percentile interval: 19 345–26 003) to 21 696 (95% percentile interval: 19 690–26 446) by August 2022 (Table 2). Whereas in the no COVID-19 plus PrEP scenario, the number of MSM with HIV was intermediate to the estimates of the other two scenarios, at 21 590 (95% percentile interval: 19 617–26 329) (Table 2). Further details are provided in the Supplementary Material.

### Impacts of each time series variable on the HIV epidemic

Our model identified sexual partners as the biggest driver of the changes in new HIV infections (Table S3, Figure S2), with a reduction of 24.9% (95% percentile interval: 20.7–26.5) over the simulation period compared to the no COVID-19 scenario. Other variables had smaller effects: HIV testing (2.5% increase, 95% percentile interval: 0.7–3.2), condom use (1.7% reduction, 95% percentile interval: 0.7–4.3), PrEP use (2.5% increase, 95% percentile interval: 1.5–3.0), and migration (0.2% increase, 95% percentile interval: -0.4 to 1.1). From January 2020 to August 2022, the observed 20.6% reduction in the number of sexual partners led to a decrease of 314 (95% percentile interval: 207–405) new HIV infections, compared to the 13.7% reduction in HIV testing which led to an increase of 31 (95% percentile interval: 8–41) new infections.

Our model also identified sexual partners and HIV testing as the major drivers of the substantial decrease in HIV diagnoses over the study period, with contributions of 10.6% reduction (95% percentile interval: 6.0–19.0) and 7.5% reduction (95% percentile interval: 7.2–8.2), respectively (Table S3). The observed 20.6% reduction in the number of sexual partners and 13.7% reduction in HIV testing led to decreases of 170 (95% percentile interval: 115–217) and 121 (95% percentile interval: 71–185) new HIV diagnoses, respectively.

For age-stratified analyses, the reduction in sexual partners among older MSM (≥50 years) contributed to a larger decrease in new HIV infections (31.0% reduction, 95% percentile interval: 26.7–32.6) compared to younger MSM (<50 years) (19.9% reduction, 95% percentile interval: 15.6–21.8) (Table S3). Similarly, the decrease in HIV testing had a larger impact on HIV diagnoses among older MSM (15.5% reduction, 95% percentile interval: 14.9–16.5) than younger MSM (2.6% reduction, 95% percentile interval: 2.2–3.4). See Supplementary Material for further details.

## Discussion

Our study is the first study to incorporate the time-varying effects of COVID-19 restrictions on the HIV epidemic among MSM using real-world data. We used a mathematical model to evaluate the impact of COVID-19 on the HIV epidemic among MSM in Australia. COVID-19-related interruptions to HIV testing, PrEP use, and migration, as well as the associated impact on partner numbers and condom use, resulted in a substantial decrease in both diagnoses (18.7%) and new infections (27.6%). The largest reduction in new infections (44.2%) occurred in 2020, followed by a rebound in 2021 and then a reduction to a stable level in 2022 (similar to the level estimated if COVID-19 had not occurred). This suggests that the direct impact of COVID-19 on HIV transmission dynamics may have diminished over time. In contrast to previous studies [13–16], our results show the temporal impacts of COVID-19 on the HIV epidemic by capturing monthly changes in sexual partners, HIV testing, condom use, PrEP use, and migration in real-world situations.

Our study identified reductions in sexual partners and HIV testing as the primary drivers behind the observed decline in HIV diagnoses, which highlights the importance of maintaining HIV testing services. Telehealth-delivered, web-based, and home-based HIV testing services could be promoted to ensure continuing testing [31–33]. This is particularly important given the potential for lag effects, as evidenced by an increasing trend in the proportion of late diagnoses among MSM between 2019 and 2022 [12]. Additionally, the age-stratified analyses in our study found that the decrease in HIV testing had a larger impact on HIV diagnoses among older MSM than younger MSM. This may lead to more late diagnoses among the older population, a trend that has also been observed [12].

We found that physical distancing restrictions and the resulting reduction in sexual partners had the biggest impact on new infections, with a 24.9% decrease alone compared to a 27.6% reduction when all changes were included, counteracting the increased risk of infection due to disrupted HIV testing and PrEP use. Our findings are consistent with previous modeling. A study from the United States also found that sexual partner numbers were the biggest driver of the change in new HIV infections, and a theoretical 50% decrease in the sexual partner numbers could reduce the number of new HIV infections by 24% [14]. Another study showed that a reduction in sexual partners could offset the impact of HIV service disruptions [34]. However, these studies used theoretical scenarios and did not consider empirically observed changes. In contrast, our study incorporated data on the actual dynamics of these time-sensitive factors in real-world situations. Additionally, our age-stratified analyses found that the reduction in sexual partners among older MSM led to a larger decrease in new HIV infections compared to younger MSM, suggesting that older MSM may have adhered more strictly to physical distancing measures or experienced greater changes in sexual networking during the pandemic.

This study projected that continued PrEP scale-up would have slightly reduced the number of new HIV infections with an intensified impact over time, which is consistent with findings from previous studies [35,36]. This finding highlights the importance of the continuity and expansion of PrEP use. Despite the COVID-19 pandemic disrupting the increasing trend, PrEP use had fully recovered by October 2021 and has continued to increase steadily through to December 2023 [37].

There are several limitations in our study. First, the simplicity of the model should encourage caution when interpreting the results. However, our validation shows the model estimates monthly HIV diagnoses well, and the robustness of the time series data for PrEP use, HIV testing, condom use, and sexual partners supports the reliability of the results. Second, we did not have monthly data on ART use and viral suppression to incorporate into the model. While reductions in ART use and viral suppression will affect transmission [14–16], access to ART essentially continued uninterrupted in Australia, even during the lockdown restrictions [38,39]. This finding suggests that the uncertainty introduced by the lack of monthly data on ART use and viral suppression is likely minimal. Third, we did not have data for the key variables during March and April 2020, as the Flux study only started in May 2020. We assumed that the values in April 2020 were the same as for May 2020, and the impact on these variables for March was half of that in April (given that restrictions and the border closure began in mid-March). As some restrictions were eased in May 2020, our model may underestimate the impact of restrictions during March and April 2020 [40]. Fourth, we did not conduct further subgroup analyses to capture heterogeneity by residential region, socioeconomic status, ethnicity, or other demographic factors beyond age, as the available data did not have these granularities. Lockdown measures varied significantly across states and territories, and socioeconomic and cultural factors may have influenced adherence to restrictions and access to HIV services. Future analyses could explore jurisdictional and demographic variations. Fifth, potential discrepancies arise because we used the proportion of MSM with casual partners reporting no condomless anal intercourse in the past 4 weeks and the number of sexual partners as proxies for condom use and overall sexual activity. Sixth, in our model, we assumed that changes in condom use and PrEP use are independent, which does not reflect that those taking PrEP are generally also reducing condom use. By applying the relative risks independently, the model may overestimate HIV transmission risk and underestimate the impact of PrEP use. Similarly, we assumed that PrEP use in HIV-negative MSM is independent of ART and viral suppression status in their partners. Again, this may underestimate the impact of changes in PrEP use if PrEP users are more likely to have partners with unsuppressed HIV viral load. Finally, our study attributes the change in factors related to HIV transmission to COVID-19 restrictions, whereas some changes might have occurred independently. While there is strong evidence of COVID-19-related disruptions given the decrease in PrEP, sexual partners, and HIV testing during the restriction periods (Supplementary Material), we cannot completely isolate the impacts from other concurrent societal changes or preexisting trends. Future studies could explore and distinguish COVID-19-specific impacts from other temporal trends.

## Conclusion

A substantial reduction in new HIV infections and diagnoses in Australia occurred during the early stages of the COVID-19 pandemic, largely due to reduced sexual partnerships. A rebound in infections between 2021 and 2022 highlights the imperative to maintain vigorous response efforts and take advantage of the gains made to virtually eliminate HIV transmission in Australia. This outcome shows how changes in time-sensitive factors in response to large-scale public health policies or interventions can affect HIV epidemics. By understanding this relationship, public health authorities can better prepare for and mitigate the effects of similar disruptions on the HIV epidemic in the future.

## Acknowledgements

R.W. is supported by an Australian Government Research Training Program Scholarship. The Kirby Institute is funded by the Australian Government Department of Health and is affiliated with the Faculty of Medicine, UNSW Sydney, Australia.

R.W. and R.T.G. conceived the study. R.W. and R.T.G. developed the model. R.W. set up and ran the scenarios. R.W. drafted the manuscript. J.A.K., M.H., S.M., and R.T.G. validated the model input. All authors were involved in writing and revising the manuscript and approving the final version.

This manuscript was previously posted to medRxiv: doi: https://doi.org/10.1101/2024.12.15.24318054

### Conflicts of interest

There are no conflicts of interest.

## Supplementary Material

Supplemental Digital Content
